# Safe Periods and Safe Activities: Two Phenological Responses to Mortality

**DOI:** 10.1002/ece3.70718

**Published:** 2025-02-02

**Authors:** Théo Constant, F. Stephen Dobson, Sylvain Giroud, Caroline Habold

**Affiliations:** ^1^ UMR 7178, Centre National de la Recherche Scientifique, Institut Pluridisciplinaire Hubert CURIEN Université de Strasbourg Strasbourg France; ^2^ Department of Biological Sciences Auburn University Auburn Alabama USA; ^3^ Department of Interdisciplinary Life Sciences, Research Institute of Wildlife Ecology University of Veterinary Medicine Vienna Vienna Austria; ^4^ Energetic Lab, Department of Biology Northern Michigan University Marquette Michigan USA

**Keywords:** hibernation, life history theory, migration, phenology, predation, reproduction, survival

## Abstract

Phenology is often thought to evolve mainly in response to food availability, yet recent studies have focused on predation. Predation may explain apparent mismatches between phenology and resources. One type of phenological response to predation involves shifting phenology from a period of high to low predation (i.e., a safe‐period strategy). This strategy presupposes variation in predation over time due to environmental factors such as the number or diversity of predators. Predation varies not only over time but also among different activities like reproduction and dormancy. Alternative activities involve alternative behavioral or physiological states, and different locations where they take place influencing predation risk. Phenological responses to predation may involve shifting from a high risk activity to a safer one, resulting in increased survival (i.e., a «safe‐activity» strategy). This strategy may theoretically evolve under environmental conditions associated with constant predation over time, but assumes variation in predation among activities. Safe‐period and safe‐activity strategies are not mutually exclusive, but assume different conditions for their evolution. On the basis of a literature review, our goal was to: (1) propose a classification of phenological responses to predation according to their evolutionary context, including mean population responses and interindividual differences (degree of synchrony); (2) to show how these two strategies may explain the lack of support for the idea that phenology responds primarily to food availability; and (3) to propose several approaches for testing the influence of predation on phenology. Our review highlights the relevance of studying phenology on multiple scales, thereby integrating several interspecific interactions (communities scales) and multiple activities (annual scale), and studying synchronicity and the pace‐of‐life (inter‐individual scale).

## Graphical abstract

Phenology is thought to have evolved mainly in response to resource availability, yet recent studies show contradicting results. Predation may explain a mismatch between phenology and the most favorable conditions with regard to resources. Phenological shifts may thus be also considered an adaptive response to predation.

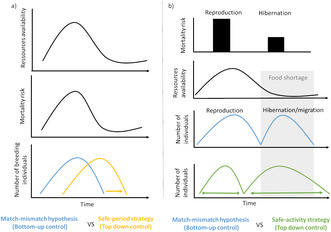

## 1 Introduction

Bottom‐up control of natural populations, that is, control of numbers by lower trophic levels such as food resources, has long been considered the most important effect underlying demographic vital rates, population dynamics, competition, and community structure (Lack 1954; Leibold 1989; White 2008). Thus, natural selection should favor phenological timing of life events by consumers that predictably matches the phenology of food resources. The match–mismatch hypothesis focuses on the synchrony between the peak of annual consumers' energy demand during reproduction and the peak of annual food supply, since changes in synchrony are supposed to impact fitness (Cushing 1973). The basic idea is that an adaptive “match” is favored by natural selection, and any “mismatch” is maladaptive. However, recent analyses show a lack of evidence for the match–mismatch hypothesis, at least in terrestrial systems (Kharouba and Wolkovich 2023, 2020). Similarly, dormancy is considered an adaptation to survive periods of energy shortage. However, phenology often does not closely match these periods (Constant et al. 2024). Such results challenge the prevailing idea of a major influence of energy resources on the evolution of phenology.

A nonmutually exclusive alternative to energetic limitations on phenologies is that extrinsic mortality constitutes a reason for mismatches between consumers and their annual food supplies. Individuals may reproduce in less favorable conditions from an energetic point of view, and thus potentially impact their reproductive success to the benefit of lower mortality. Among sources of mortality, predation (a “top‐down control,” from a trophic viewpoint) is likely an important species interaction that influences population dynamics (Salo et al. 2010). Thus, phenology may be determined by both top‐down and bottom‐up influences, and they may interact synergistically (selection for the same period) or antagonistically (selection for different periods). It is thus necessary to consider the diversity of phenological responses to mortality, particularly from predation.

**FIGURE 1:**
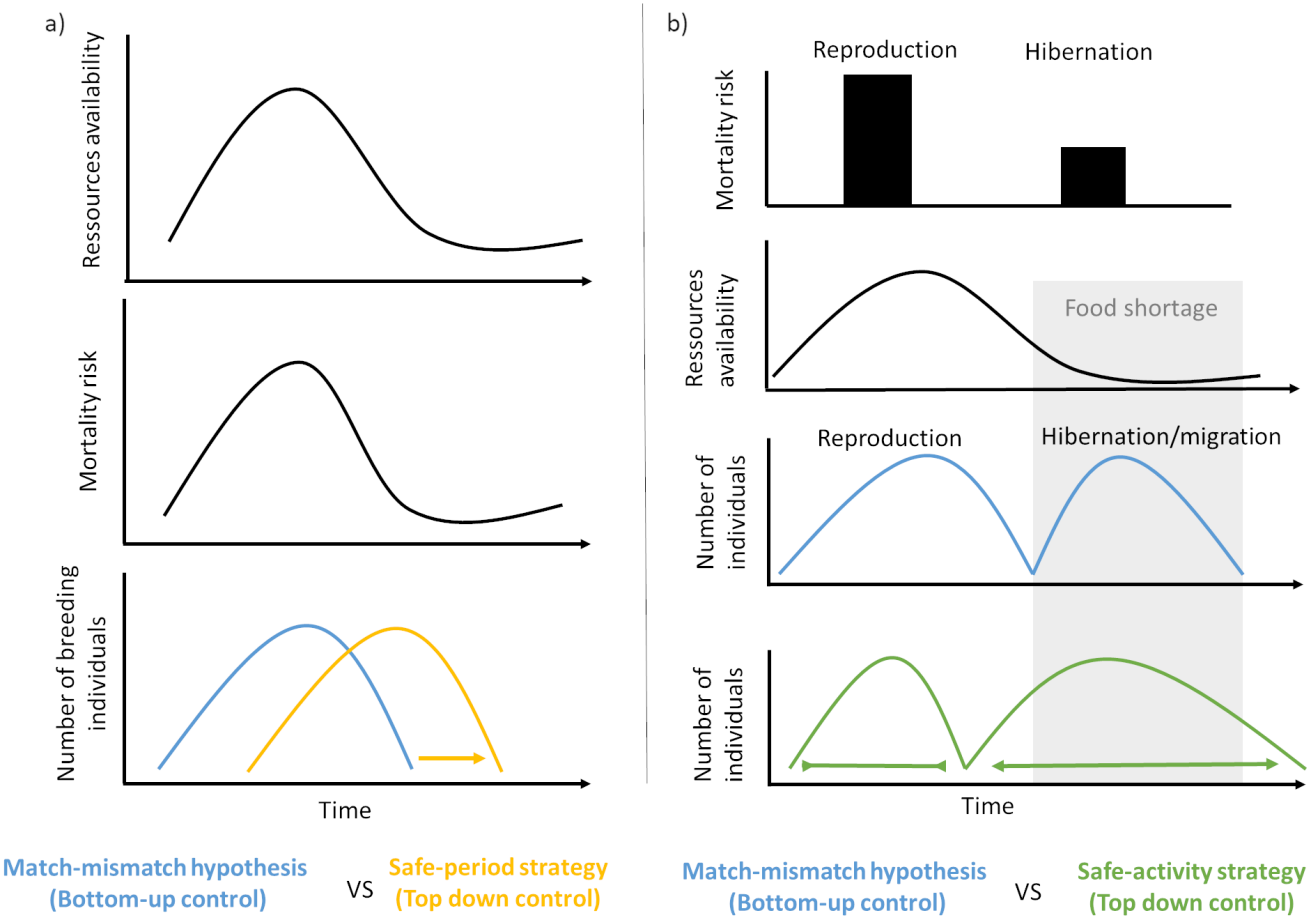
Schematic representation of the phenology of activities according to the hypothesis of top‐down or bottom‐up influences. (a) According to the match–mismatch hypothesis, the phenology of consumers should evolve according to the phenology of resources. In this case, reproduction should occur during the period of high food availability. On the contrary, the safe‐period strategy proposes a phenological shift toward the period of low predation risk. (b) According to the match–mismatch hypothesis, hibernation or migration is supposed to occur during the period of low food availability and reproduction during the period of high food availability. On the contrary, the safe‐activity strategy proposes a limit to the time spent in risky activities such as reproduction and spending more time in a safe activity such as hibernation. This means that hibernators become inactive while the conditions are still favorable for remaining active (Constant et al. 2024). The duration of the safe‐activity strategy varies according to the trade‐off between survival and reproduction. Contrary to the figure, the end of the safe activity strategy may also be during the food shortage period to increase reproductive success (see the example of hibernating males). Arrows show the direction of phenological shift for safe‐period and safe‐activity strategies. The abbreviation “vs.” stands for versus, that is, in comparison with.

We classified two categories of phenological shifts in response to mortality: the “safe‐period” and “safe‐activity” strategies. We examined “phenological shifts,” in comparison to what would be expected if phenology was solely explained by energetic considerations (termed the “energy model”), which we consider to be a theoretical baseline. However, predation, food availability, and other factors act simultaneously to shape phenology. One strategy is a phenological shift from a period of high to reduced mortality, along with an increase in survival of adults or their offspring (i.e., the safe‐period strategy; Figure 1). The safe‐period strategy has already been studied in the context of match–mismatch hypothesis (Hušek et al. 2012; Nakazawa and Doi 2012; Reneerkens et al. 2016), and in the context of predation (Götmark 2002; Reneerkens et al. 2016) or competition (Dumandan, Yenni, and Ernest 2023; Dyugmedzhiev, Slavchev, and Naumov 2019). This strategy presupposes a variation in mortality over time for a given activity in a singular location.

Mortality varies not only over time for a given activity but also among activities, depending on morphological, behavioral, or physiological characteristics and location. For example, reproduction is a high‐risk activity for prey due to intense physiological effort and exposure to predators (Magnhagen 1991). In contrast, dormancy is a safe activity, since animals remain inactive for several months in a shelter (Constant et al. 2020; Turbill, Bieber, and Ruf 2011). Thus, a second strategy is a phenological shift from a high‐risk activity to a safer activity to increase survival. This strategy might evolve in environments with constant or variable predation but assumes high variation in predation among activities, at least for part of the year. We refer to this as the “safe‐activity strategy” (Figure 1). Dormant or migratory species seem to be examples of the safe‐activity strategy as they show high differences in mortality among activities.

On the basis of a literature review, our goal is threefold. (1) We propose a classification of phenological responses to mortality according to their evolutionary context. Initially, we address the evolutionary similarities of phenological responses to mortality. We propose explanations of when, where, and by which species and individuals alternative phenological strategies may occur. (2) We examine the specificities of each safe strategy. We discuss the safe‐period strategy in which we include the average population response and synchronicity of individuals within a population. We also present evidence for the safe‐activity strategy, a previously understudied concept. For each strategy, we present case studies and demonstrate how these two strategies may explain the lack of support for the energy model. (3) We propose several approaches for testing the influence of mortality on phenology based on evolutionary principles. To illustrate the logic behind these two strategies, we take the example of predation, which is assumed to be an important and the most studied mortality factor in this context, but these strategies can be adapted to other sources of mortality, such as competition and disease. The aim of our review is to highlight the relevance of studying phenology at multiple scales, whether by integrating interspecific interactions (community scale), multiple activities (annual scale), or by studying synchronicity and the pace of life (interindividual scale).

## 2 Evolutionary Similarities of Phenological Responses to Predation

### 2.1 When?

Phenological shifts (relative to energy model) in response to predation may be associated with two types of trade‐offs. The first is that time may be shared between activities that influence different fitness components (i.e., activity trade‐offs). For example, a trade‐off between remaining in reproductive mode, to increase reproductive success, versus entering dormancy to increase survival. In the absence of an activity trade‐off, that is, in the absence of an alternative activity with equivalent fitness gain, phenology may be subject to a trade‐off between periods that influence different fitness components (i.e., a period trade‐off). For example, a trade‐off between a period with high reproductive success but high mortality risk, and a period with low reproductive success and low mortality risk. The safe‐period and safe‐activity strategies may therefore be evolutionary responses that favor survival in the context of activity and period trade‐offs. These trade‐offs may explain both the beginning and the end of the safe period and activity. The beginning is explained by a higher survival benefit, and the end may be explained by higher benefits in terms of reproductive success or growth. Without trade‐offs, the phenology under a safe‐period and safe‐activity strategy is explained by the advantage of the survival benefit.

Trade‐offs of survival and reproductive success may vary according to age, gender, species, and other variables (endogenous and environmental) that change within individuals across time (e.g., energetic reserves and weather), hence the evolution of state‐dependent life histories (including state‐dependent phenologies). We therefore expect variations in the phenology of safe strategies at these different scales due to variations in lifecycle trade‐offs (see specific examples below).

### 2.2 Where?

The benefits of both the safe‐period and safe‐activity strategies increase with predation pressure. From an extrinsic point of view, predation pressure is assumed to be greater at lower latitudes due to a disproportionate number of predator species (Schemske et al. 2009), but varies on a small scale depending on habitats (Large and Smee 2013) and predator composition (Finke and Denno 2005). The primary constraint of a phenological shift relative to the peak of food availability may be energy constraints (see section “Lack of support for the match‐mismatch hypothesis”). The energetic cost of phenological shifts may increase with strong temporal constraints, as in a highly seasonal environment. In such environments, species have very short time spans when conditions are favorable for carrying out essential activities. For instance, many adult endotherms and ectotherms must reach a particular developmental stage or accumulate sufficient energy reserves before dormancy or migration is possible (Olsen, Thum, and Rohner 2021). Furthermore, adults must reproduce in time for the young to grow and accumulate reserves for their initial dormancy or migration. We can expect that the extent of the phenology shift (in relation to food availability peak) will decrease with strong seasonality and extended essential activities. In addition to seasonality, harsh environmental conditions may also induce time constraints and limit the use of these strategies. In some hibernators, males immerge earlier than females, probably to escape predators. However, the gap between males and females decreases for species living in environments with low annual precipitation, probably because low food and water availability delays postreproductive recovery of body condition, and thus the beginning of hibernation (Constant et al. 2024).

### 2.3 Which Species or Individuals?

The safe‐period and safe‐activity strategies are both predator avoidance strategies (see “safe‐period” below for an exception) since they limit the probability of encountering predators (Heithaus et al. 2009). Thus, species that already possess antipredation behaviors or predator escape traits might be less likely to exhibit phenological shifts as a predator avoidance strategy. More specifically, the evolution of an antipredation strategy may trade‐off with the evolution of predator avoidance strategies. Thus, if a prey no longer encounters a predator while active, then there may not be sufficient selection pressure to develop or maintain antipredation mechanisms (Brodie, Formanowicz, and Brodie 1991). For example, species of fish or reptiles that have morphological traits that provide defense against predators appeared to reduce use of refuges or escape behaviors compared to species that do not have such antipredation defenses (Blanchard and Moreau 2017; Losos et al. 2002). Similarly, a species that already uses a predator avoidance strategy is theoretically less likely to develop another one, if predation pressure is sufficiently reduced. Antipredation and predator avoidance strategies include the evolution of large size, flight, arboreality (e.g., Shattuck and Williams 2010), fossoriality, chemical protection (e.g., Blanco and Sherman 2005), eusociality (e.g., Keller and Genoud 1997), or hibernation (Turbill, Bieber, and Ruf 2011).

However, there are a few conditions under which phenological shifts may be found in addition to other antipredation or predator avoidance traits. First, in the case of reproduction, antipredation and predator avoidance strategies may be effective for adults, but not offspring. For example, many birds show a phenological shift during breeding despite their ability to escape predators by flight (Hušek et al. 2012; Reneerkens et al. 2016). In this case, phenological shift is effective in increasing the survival chances of clutches that cannot defend themselves. Second, species that usually encounter a variety of predators may have different antipredation or escape strategies for each of them. This may explain why some species exhibit multiple defenses (Kikuchi et al. 2023). Finally, if the strategy of antipredation behavior or predator avoidance is limited in periods such as hibernation or migration, then a phenological shift may increase annual survival by spending more time on this activity (i.e., a safe‐activity strategy).

The safe‐activity and safe‐period strategies should be expected in species, populations, genders, or individuals with slower life‐history strategies (those with greater survival and more limited periodic reproduction; Stearns 1989, 1992), for which survival benefits have a greater effect on fitness (Iler et al. 2021). The safe‐activity and safe‐period strategies may also be associated with behavioral, physiological, and hormonal traits that favor survival. The pace‐of‐life syndrome (POLS) is an extended idea of life‐history theory that attempts to explain the covariation of phenotypic traits among species, populations, and individuals (Réale et al. 2010; Ricklefs and Wikelski 2002). The POLS adds other biological traits, such as physiological, hormonal, and behavioral attributes, that show similar trade‐offs along the slow–fast continuum (Réale et al. 2010). For example, a population with a slow life history might have risk‐averse behaviors, avoidance of oxidative stress, and strong immune responses. The POLS can also be applied to the maintenance of interindividual differences within populations (Réale et al. 2010). In this case, the range of individual phenology within a population may be viewed as a continuum, with each extreme of the continuum associated with a fast or a slow pace of life. Individuals at the slow end of the continuum may show a safe‐period or a safe‐activity strategy.

## 3 The Safe‐Period Strategy

### 3.1 Mean Population Response

The safe‐period strategy involves phenological shift from a high‐ to a lower‐risk predation period. Fitness benefits are expected to increase with the difference in survival between the periods of high and low predation risk. Therefore, an essential prerequisite for this strategy is annual variation in predation for a given activity and location (Figure 1). This variation may be attributed to changes in the number or diversity of predators (Grant et al. 2005a), predator behaviors (Burhans et al. 2002; Wilson, Martin, and Hannon 2007), or alternative prey availability (Nordberg and Schwarzkopf 2019). Thus, pulses in resource availability and the departure and arrival of dormant and migratory species (as prey or predator) contribute to a high annual variability in predation risk in seasonal habitats (Holyoak, Caspi, and Redosh 2020; Sperry et al. 2008). The effectiveness of predator hunting techniques may also vary seasonally due to environmental conditions (reviewed by Varpe 2017).

Survival benefits of a safe‐period strategy are greater for potentially risky activities. Thus, safe‐period strategy should be observed with reproduction in seasonal environments, but limited by time constraints. Reproduction is assumed to be a period of high risk to predation, often due to decreased activities of predator detection and escape capabilities (due to increased activity in finding partners, using visual, olfactory, and vocal signals; Magnhagen 1991). In mammals, for example, pregnant females are bulkier and thus may be less able to escape predator attacks. Predation risk is also increased for species in which males enter intense competition, as they may suffer injury, reduced physical condition, and isolation from a larger social group (Owen‐Smith 2008). In general, newborns and juveniles are particularly at risk of predators compared to adults (Giachetti et al. 2022; Longland and Jenkins 1987). Several examples of safe‐period strategies show a phenological shift of reproduction in response to predation compared to what would be expected from an energetic perspective (Hušek et al. 2012; Reneerkens et al. 2016). For example, high nesting predation during the breeding season can lead to delays (Hušek et al. 2012; Reneerkens et al. 2016) or advances (Götmark 2002) in laying dates in relation to food peaks. In the beetle *Ellychnia corrusca* (winter firefly), reproduction takes place in spring, perhaps to escape specialist predators that are active in the summer (Deyrup et al. 2017).

Another example of a safe‐period strategy appears with migration. Several birds show a shift in migration phenology that promotes predator avoidance during travel (Jonker, Eichhorn, and Bauer 2010), on the breeding grounds (Harts, Kristensen, and Kokko 2016), or in overwintering areas (Lank et al. 2003; Ydenberg 2022). For the Western sandpipers ( *Calidris mauri* ), departure from the breeding grounds seems to deviate from the energy model as no decrease in food availability occurs on the breeding grounds or at stopover sites, even if a general influence of food on the migration phenology of this species cannot be ruled out (Lank et al. 2003). For several species, migration phenology is probably explained both by predator avoidance (during travel or at the arrival site) and by food availability (Jonker, Eichhorn, and Bauer 2010; Lank et al. 2003). Migration phenology may be explained by the safe‐period strategy but also the safe‐activity strategy (see below for details).

A phenological shift of reproduction may be associated with reduced reproductive success (Reneerkens et al. 2016). Thus, there may be a trade‐off between period with high reproductive success but low survival rate, and a period with low reproductive success and high survival rate (i.e., a “safe‐period” trade‐off). In this case, the safe‐period strategy may be associated with a slowing of the POLS, as well as explaining interindividual differences. For example, in Eurasian blackbirds ( *Turdus merula* ), the exploratory character of individuals, breeding phenology, and reproductive success seemed to be associated, depending on perceived predation levels (Abbey‐Lee and Dingemanse 2019).

### 3.2 Synchrony

Predation rates are not only dependent on predator density but also on prey density (Oaten and Murdoch 1975). For example, an increase in predation was observed with the arrival of migratory species (Bestley et al. 2010; Madsen and Shine 1996) and the emergence of dormant species (Grant et al. 2005b; Sperry et al. 2010). The degree of synchrony of individual phenologies within a population may produce increased or decreased predation as prey density changes. A strong synchrony may be observed at different stages of reproduction, such as the date of emergence from dormancy for adult cicadas, leading to the start of reproduction (Blackwood et al. 2018), or the date of hatching for turtles (Santos et al. 2016).

Several hypotheses might explain a reduction in predation as a result of synchrony (Ims 1990a). Breeding in the same place at the same time leads to the formation of a large group, which (1) causes a satiation effect, as predators are limited in the amount of prey they can ingest, (2) increases detection of predators, and (3) confuses predators, making it harder to choose specific prey (reviewed by Fairbanks and Dobson 2007). Thus, synchrony seems to be an antipredation strategy that reduces the probability of a successful attack when encountering a predator (see dilution effect in Lehtonen and Jaatinen 2016). At another extreme, it has also been suggested that a strong asynchrony may limit predation by facilitating avoidance of predators, especially if it takes time for predators to improve their hunting techniques or focus on a specific prey item after prey density reaches a particular abundance (Sinclair, Mduma, and Arcese 2000). We assume that asynchrony and synchrony are part of the safe‐period strategy, as either should result in phenological shift from a high‐ to a lower‐risk predation period.

The energy model predicts synchrony of phenology in highly seasonal environments, where only a small part of the year provides sufficient food for reproduction (Ims 1990a). In this case, natural selection favors a similar phenology of reproduction among individuals via bottom‐up environmental factors. However, a higher synchrony than expected from environmental effects is sometimes observed. In environments with low seasonality, synchrony is suggested to reduce predation risk (Ims 1990a). Theoretically, although mean phenology might be explained by bottom‐up influences, the variance in phenology (or synchrony) might be explained by a top‐down process. On the other hand, asynchrony may occur in an environment where the bottom‐up influence on phenology is relatively weak, as when food is available during a large part of the year.

Many hypotheses have been formulated to explain the evolution of synchrony and asynchrony of phenology in response to predators. It has been suggested that asynchrony is more beneficial (1) for species that are relatively low in abundance compared to predators, and therefore do not induce satiation (Sinclair, Mduma, and Arcese 2000); and (2) against generalist predators that would switch from one prey species to another according to their density (Ims 1990b). On the contrary, synchrony would better suit specialist predators that reach satiation (Ims 1990b). Although these hypotheses have been studied (Descamps 2019; Michel et al. 2020; Sinclair, Mduma, and Arcese 2000), no consensus of general support has yet occurred. A prerequisite for this strategy is that variation in conspecific density during activity is likely to change the predation risk, but selection pressures differ between synchrony and asynchrony. Evolution of synchrony should be favored when the highest predation occurs before and after the mean date of reproduction. Around the mean date of reproduction, individual density is high enough to induce satiation of predators, but before and after this period, prey density is not sufficient to “swamp” predators, and prey are subject to a relatively higher rate of predation (Michel et al. 2020). On the contrary, asynchrony should be favored by natural selection when individuals that reproduce over a short period are more likely to experience predation. These individuals should be more easily detected by generalist predators (Sinclair, Mduma, and Arcese 2000).

## 4 The Safe‐Activity Strategy

### 4.1 Specific Features

The safe‐activity strategy consists of a phenological shift (compared to energy model) from an activity associated with high risk, such as reproduction, to an activity associated with low risk, such as dormancy or migration, thus increasing survival (Figure 1). The difference in predation risk among activities may be explained by: (1) morphological, behavioral, or physiological changes that reduce the probability of detection by a predator, and (2) a change to a location with limited detection, such as burrows, or with fewer predators. The case of dormancy is thus associated with changes in behavior, physiology, and location (e.g., in a hibernaculum, such as a burrow or cave). Contrary to the safe‐period strategy, the effectiveness of the safe‐activity strategy is not dependent on environmental variation in predation but depends on a variation in mortality risk between activities. In theory, the safe‐activity strategy may therefore be less restricted to seasonal environments. In practice, dormancy and migration are indeed present all over the globe, although they are more present in seasonal environments (Somveille et al. 2013; Wilsterman, Ballinger, and Williams 2021). This pattern undoubtedly reflects the fact that these traits serve not only as means of predation avoidance but also allow animals to avoid environmental unsuitability across space and time.

**FIGURE 2:**
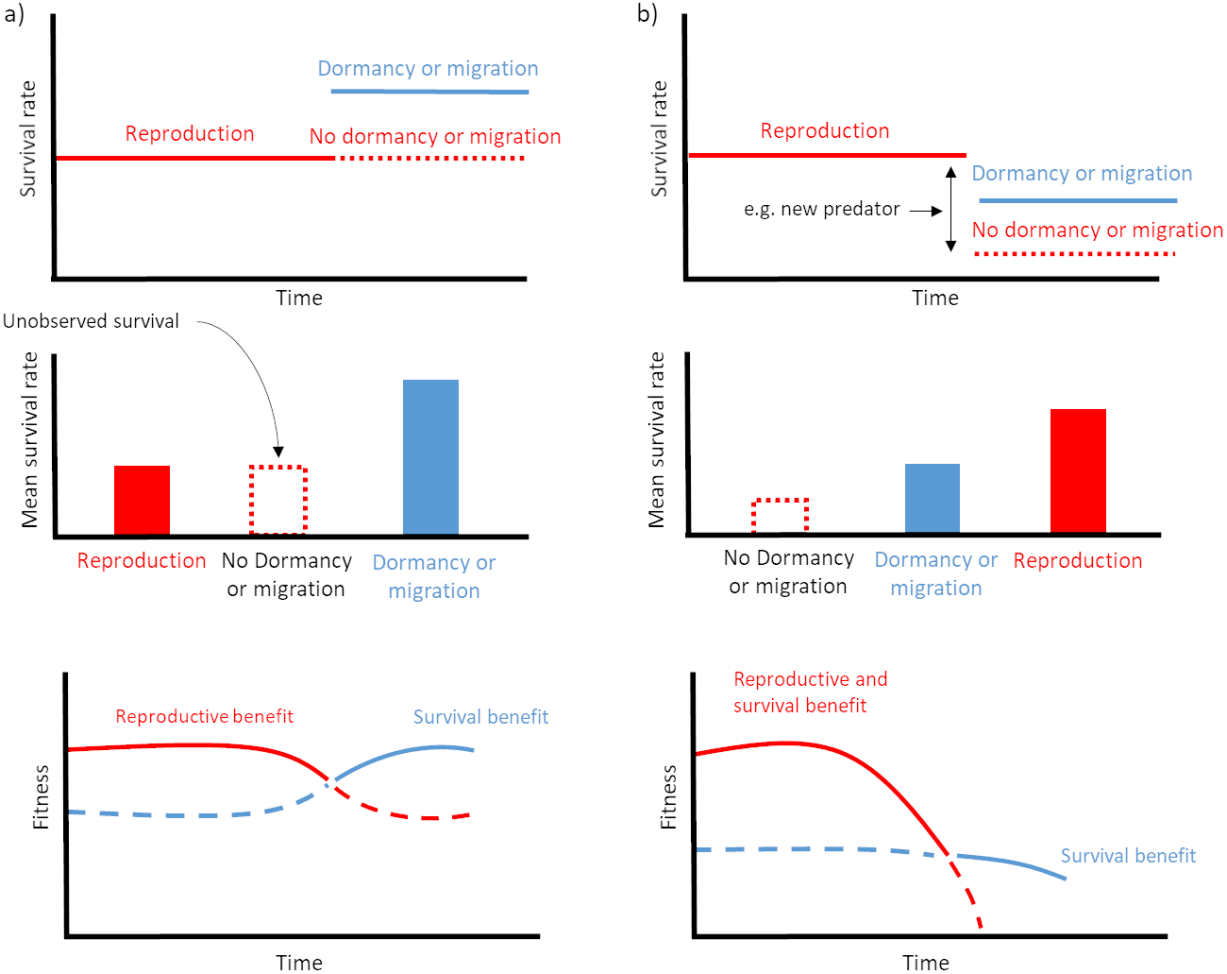
Schematic representation of annual variation in mortality risk and fitness in the case of (a) high and (b) low survival rate during the safe activity. The dotted lines and rectangles represent the theoretical survival rate and fitness if animals were in hibernation, migration or reproduction at a time of year when they are not engaged in these activities.

Identifying safe activity may be evident if survival during this activity is close to 100%, as in the case of certain dormant species (see below). When the survival rate is lower, identification can be more complex. The prerequisite of a safe activity is an increase in fitness compared with the option of remaining in a riskier activity. However, if the safe activity has been selected, the fitness benefit of the other option is not measurable. Comparing the average survival rate between activities in the annual cycle might be an indicator. However, a safe activity may lead to a decrease in survival if other options would lead to a more significant decrease (Figure 2a,b). It is therefore necessary to focus on multiple clues to identify safe activities. We have identified several clues that we used to identify migration and dormancy as a safe strategy: to measure the effect on fitness or life history of (1) interindividual and interspecific variations in the safe‐activity phenology, (2) variations in the predation rate or in the perception of the risk of predation in nature or through experiments (see section “Testing predation's effect on phenology”), and (3) identification of a phenological shift in comparison to the energy model with an increase in survival.

The complexity of identifying a safe activity lies partly in the fact that some activities may increase survival only in particular periods of the annual cycle (Figure 2a,b). For example, dormancy may be constrained by certain environmental conditions such as cold temperatures, or limited by the amount of reserves that can be stored. Variations in the survival benefits of a safe activity over time may therefore limit the safe‐activity strategy. In addition, anthropogenic change may reduce the benefits of such a strategy, for example, with reduced migration survival (Palacín et al. 2017) or higher energy costs for hibernation (Chmura et al. 2023).

Although the safe‐period and safe‐activity strategies evolve from different conditions, they are not mutually exclusive and may combine to reduce predation. For example, extended dormancy or migration period (travel and residence) may combine with strong synchrony at emergence or arrival at breeding grounds, both reducing predation. Similarly, individuals may benefit both from shifting reproduction to a safer period and reducing reproduction duration to increase the time devoted to a safer activity. Future studies are needed to test for the presence of such dual strategies.

### 4.2 Dormancy

Dormancy (used as general descriptor hereafter) includes hibernation and diapause and characterizes prolonged inactivity of several months associated with phases of hypometabolism and hypothermia. This type of life history is found across the animal kingdom (Wilsterman, Ballinger, and Williams 2021). Dormancy appears to be particularly effective in reducing extrinsic sources of mortality, such as predation. Dormancy in many ectotherms and endotherms is associated with a high survival rate when compared to the active season since dormant individuals are hidden for several months in places protected from predators (Tanner and Jorgensen 1963; Turbill, Bieber, and Ruf 2011; Wilson and Cooke 2004). Dormancy increases longevity at both intra‐ and interspecific levels, compared to nondormant species of similar size (Magombedze, Ferguson, and Ghani 2018; Turbill, Bieber, and Ruf 2011; Wiklund, Gotthard, and Nylin 2003). In some species, the survival rate over several months of dormancy is close to 100% within a population (Litzgus et al. 1999; Tanner and Jorgensen 1963; Turbill, Bieber, and Ruf 2011). Smaller species are those that show the highest survival benefit from dormancy when compared to nonhibernating species of the same size (Turbill, Bieber, and Ruf 2011). This increased benefit may be explained by the fact that smaller species are presumed to suffer higher predation rates during the active season (Cohen et al. 1993).

Survival benefits from energy shortage and other mortality risks during the period of dormancy may influence phenology. In various taxa, increased energy constraints along latitudinal and altitudinal gradients (e.g., associated with a decrease in primary productivity and temperature) are accompanied by an increase in dormancy duration (Turbill and Prior 2016; Wilsterman, Ballinger, and Williams 2021). Thus, energy constraints are assumed to influence at least part of the dormancy period. Nevertheless, other evidence suggests that dormancy phenology is not initiated solely in response to deficiencies in energy, water, or poor food quality. Several observations of both heterothermic endotherms and ectotherms suggest that dormancy may occur while energetic conditions still enable activity (reviewed in Constant et al. 2020, 2024). For example, edible dormice ( *Glis glis* ) exhibit prolonged hibernation periods of 8 to 11.7 months, at times when there are no apparent energetic constraints in the environment. Indeed, the plant growing season exceeds the dormouse active season by 2 months (for an 8‐month hibernation; Bieber et al. 2014). Dormancy allows dormice to escape predators (Hoelzl et al. 2015). A similar example can be observed in insects, where females of the common brimstone butterfly (*Gonepteryx rhamni*) show delayed emergence by 3 weeks compared to males, despite favorable energetic conditions. This enables common brimstone butterfly females to avoid extrinsic mortality prior to breeding (Wiklund, Lindfors, and Forsberg 1996). Thus, a phenological shift at either the onset or termination of dormancy, as compared to the expectation of the energy model, suggests that phenology may be used as a predator avoidance strategy. Some individuals seem to allocate excess energetic reserves toward increasing the duration of hibernation under high predation pressure, thus influencing dormancy phenology (Allison, Conway, and Morris 2023; Bieber et al. 2014), as predicted by state‐dependent optimization theory (McNamara and Houston 1996).

**FIGURE 3:**
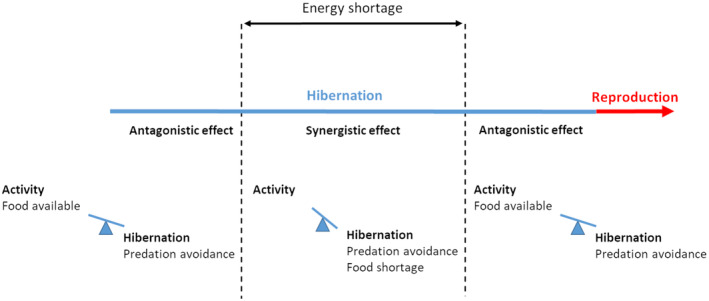
Schematic representation of the antagonistic and synergistic effect of top‐down and bottom‐up control on hibernation phenology. Hibernation is advantageous from an energetic point of view when the environment no longer enables a stable energy balance to be maintained, that is, during periods of energy shortage. Hibernation outside this period assumes that other benefits, such as predation avoidance, outweigh the benefits of activity. In this case, as energy benefits predict activity then top‐down and bottom‐up control are antagonistic. It is important to note that the figure is not to scale and is intended to illustrate the antagonistic and synergistic effects of selection forces on phenology.

Food availability and predation may act synergistically or antagonistically on dormancy phenology (Figure 3). For example, there may be a synergic effect of these two forces on dormancy phenology during periods of energy shortage, when food is not available in the environment to meet energetic needs. However, there may be an antagonistic effect of these forces on dormancy immergence and emergence. The benefits of avoiding predators favor dormancy just before and after reproduction, while food is still available in the environment. From an energetic point of view, it is more favorable to remain active as long as the environment allows a positive energy balance, as dormancy inevitably leads to energy loss.

An activity trade‐off between the benefits of survival and reproduction appears to explain age and gender differences in dormancy phenology in endotherms and ectotherms (Constant et al. 2024). In hibernators, on the one hand, the reduction of mortality due to predation may explain earlier immergence than expected from energy constraints. In some species, males immerge several weeks before females, which are still lactating for their offspring. On the other hand, reproductive benefits seem to promote early emergence, at a time when it would be more favorable from an energetic point of view to remain dormant (Constant et al. 2024). In some species, males emerge while conditions are still harsh, resulting in a loss of body mass, but this early emergence may provide benefits for reproduction. For example, hibernating male Columbian ground squirrels emerge 7–10 days before reproductive females, and exhibit increased likelihood of getting a breeding territory and increased reproductive success (e.g., Thompson et al. 2023). Female ground squirrels emerge later when vegetative food resources are more abundant (Tamian et al. 2024). On the contrary, a reduction in the trade‐off occurs when breeding takes place several weeks after female emergence, resulting in no sex difference at emergence (Graves and Duvall 1990; Olsson, Birkead, and Shine 1999). Another example of this trade‐off is the variation of dormancy phenology with age as shown in the edible dormouse. With increasing age, edible dormice spent more time breeding and less time hibernating. Older individuals were less likely to encounter favorable conditions (masting years) for reproduction in the future (Bieber, Turbill, and Ruf 2018). Thus, they spend more time breeding, despite a higher associated mortality risk, especially from predation.

### 4.3 Migration

Migration consists of an annual two‐way movement, with each migratory direction influenced by one of three primary benefits: feeding, reproduction, or refuge (Shaw 2016). Seasonal migration, called “refuge” migration, is assumed to be an annual two‐way movement between a place that is favorable for reproduction, and another place that improves the survival of adults or newborns for the rest of the year. This pattern is common in species that are highly mobile (Alerstam, Hedenström, and Åkesson 2003), primarily birds but also in insects, snakes, bats, and ungulates. Differences in survival rates are often apparent between nonreproductive and reproductive periods (lower: Leyrer et al. 2013; Robinson et al. 2020; similar: Robinson et al. 2020; Rockwell et al. 2017; higher: Buechley et al. 2021; Swift et al. 2020). Less evident is whether remaining in breeding habitats would lead to relatively greater mortality. Mortality during migratory movement appeared to vary among species, ranging from low (Conklin et al. 2017; Senner et al. 2019) to high (Rockwell et al. 2017; Rushing et al. 2017). Part of this variation could be explained by the lack of emergency stopover sites, especially when crossing geographic barriers (Senner et al. 2019). In some species, however, individuals that exhibit refuge migration show higher (Winger and Pegan 2021; Zúñiga et al. 2017), similar (Sandercock and Jaramillo 2002) or lower (Buchan et al. 2020) annual survival or longevity compared to residents. The migratory lifestyle seems to be compatible with multiple life‐history strategies (Pierce, Yanco, and Wunder 2024).

The causes of migration vary among species and ecosystems. Refuge migration is evolutionarily favored in response to seasonal changes in food availability, weather conditions, or predation risk (Shaw 2016). In both terrestrial and marine mammals, predation on adults or newborns (migratory movement to specific calving areas) is believed to be an important factor in promoting migration (Avgar, Street, and Fryxell 2014; Fryxell and Sinclair 1988; Shaw 2016). Several experiments with partial migrations of fish populations elegantly demonstrated that the influence of perceived risk of predation was important factor promoting individual migration (Brodersen et al. 2008; Hulthén et al. 2015; Skov et al. 2011). In both mammals and fish, migration can take place toward a less favorable location from an energetic point of view, but with a lowered risk of predation (Brodersen et al. 2008; Hebblewhite and Merrill 2011). The existence of a migration phenology that did not match what might be expected from an energetic point of view, consistent with the safe‐activity strategy.

As with dormancy phenology, there may be a trade‐off between spending time in the migration period (travel and residence) to increase survival, or during the breeding period to increase reproductive success (i.e., an activity trade‐off). In boreal birds, long‐distance migration resulted in higher annual survival than short‐distance migration; at the same time, the breeding period was shorter, and associated with lower clutch sizes and annual fecundity (Winger and Pegan 2021). This phenomenon is not the result of long‐distance migration taking more time, but rather suggests strategies that favor survival over reproduction (Winger et al. 2024). The activity trade‐off may also explain the integration of migration phenology into a slow POLS. In a fish species that exhibited both migratory and resident individuals in the same population, bold individuals are more likely to migrate than shy ones (Chapman et al. 2011), the latter being less at risk of predation (Dugatkin 1992). In this example, phenology was used as a safe activity strategy (a top‐down effect) by migrators, and by residents to match conditions favorable for growth and reproduction (bottom‐up effects).

## 5 Testing Predation's Effect on Phenology

Identifying predation as an ultimate cause of trait evolution can be particularly difficult. Predation may affect fitness in two ways, via a lethal and nonlethal influence (Cresswell 2008). The lethal effect can be measured using the correlation between the predation rate and variation in the distribution of phenotypic traits (e.g., phenology). Nonlethal effects correspond to the fitness costs associated with investing energy or time in anti‐predation or predator avoidance strategies (trait‐mediated interactions). For example, limiting movement and foraging to avoid the lethal effect of predators could reduce food consumption or mate encounters. Phenological shifts are examples of predator avoidance strategies that may be associated with strong nonlethal effects. Nonlethal effects are very common in predator–prey interactions and have consequences for population dynamics that may be as important as, or even more important than the lethal effects of predation (Preisser, Orrock, and Schmitz 2007; Werner and Peacor 2003). Nonlethal effects can be estimated using predation risk metrics. Moll et al. (2017) summarized 13 distinct metrics of predation risk divided into three subcategories: risky places (or long‐term risk, e.g., predators number and density), risky times (or short‐term risk, e.g., observed interaction), and habitat characteristics (e.g., visibility). The influence of predation on the evolution of phenology via a nonlethal effect may be assessed through the correlation between variation in predation risk metrics and the distribution of phenotypic traits (e.g., phenology) in the population.

The influence of nonlethal effects of predation on the evolution of phenology is much more difficult to measure. In fact, the response of prey to the risk of predation may be nonlinear and exhibit a threshold (Hazlett and Mclay 2005; Teplitsky, Plénet, and Joly 2005). Predators may induce effects that are disproportionate to their actual risk (reviewed by Cresswell 2011). Studies that test for a causal link between predation and a phenotypic trait by altering predator numbers may actually fail to produce evidence of an effect because they have not altered the perceived predation risk by the prey. Nonlethal effects are rarely studied in this regard and may be underestimated.

A complementary approach is to measure fitness advantages of response to predator effects. Selection induced by predation can be observed within and between generations of a population by a change in the distribution (e.g., mean value) of phenology over time and allele frequencies of the associated genes (Wade and Kalisz 1990). A part of the trait variation within and between generations may be explained by phenotypic plasticity. Phenotypic plasticity is a trait itself, and thus subject to selection that produces different phenotypes in response to different environmental conditions (Lane et al. 2019; Nussey, Wilson, and Brommer 2007). However, for natural selection to influence phenology, changes in phenotypic variation between generations must be associated with genetic variation (i.e., heritability), and studied by quantitative genetic methods (Lane et al. 2012, 2011). To date, few studies have investigated how much of the phenological shift in response to predation is explained by phenotypic selection or phenotypic plasticity, or which cues (e.g., predator calls) are involved in any plastic variation of phenology (e.g., Abbey‐Lee and Dingemanse 2019; Skov et al. 2010).

For testing a causal link between predation and the traits of prey, it is necessary to modify the predation and perceived predation risk in nature, and then measure the effect on the trait distribution (e.g., predator exclosure treatment, Karels et al. 2000). Because of the difficulty of setting up this type of study, most of the research of this type is carried out in controlled laboratory conditions or micro‐/mesocosms (Merilä and Hendry 2014).

## 6 Conclusion

Bottom‐up and top‐down influences may have synergistic and antagonistic effects on the evolution of phenology. We suggest that shifting phenology in response to top‐down effects may be more widespread in nature than previously thought and may be classified into two categories: safe‐activity and safe‐period strategies. These strategies are not mutually exclusive and their combination may improve predator avoidance and other sources of top‐down mortality. The influence of predation on phenology has mainly been studied while assuming the safe‐period strategy. However, we suggest that a safe‐activity hypothesis may also explain shifts in phenology. Species with increased survival during dormancy and migration are likely to be common candidates for testing the safe‐activity strategy. This might be especially true for hibernating species and long‐distance migratory birds that exhibit a slow life‐history strategy. We also provide a framework for measuring the influence of predation on phenology via evolutionary ecology methods or by identifying trade‐offs associated with phenology at the interspecific and intraspecific scales.

## Author Contributions

**Théo Constant:** conceptualization (lead), writing – original draft (equal), writing – review and editing (equal). **F. Stephen Dobson:** supervision (equal), validation (equal), writing – original draft (equal), writing – review and editing (equal). **Sylvain Giroud:** supervision (equal), validation (equal), writing – original draft (equal), writing – review and editing (equal). **Caroline Habold:** supervision (equal), validation (equal), writing – original draft (equal), writing – review and editing (equal).

## Conflicts of Interest

The authors declare no conflicts of interest.

## Data Availability

The authors have nothing to report.
